# Reduction in the incidence of cognitive impairment and related costs through an innovative health awareness programme in rural Japan

**DOI:** 10.1371/journal.pone.0311826

**Published:** 2024-10-14

**Authors:** Ayako Shoji, Kenichi Kudo, Koichi Murashita, Shigeyuki Nakaji, Ataru Igarashi

**Affiliations:** 1 Department of Health Economics and Outcomes Research, Graduate School of Pharmaceutical Sciences, The University of Tokyo, Bunkyo-ku, Tokyo, Japan; 2 Healthcare Consulting Inc., Chiyoda-ku, Tokyo, Japan; 3 Center of Healthy Aging Innovation, Hirosaki University, Hirosaki City, Aomori, Japan; 4 Integrated Clinical Care Informatics Inc., Bunkyo-ku, Tokyo, Japan; 5 Department of Social Medicine, School of Medicine, Hirosaki University, Hirosaki City, Aomori, Japan; 6 Department of Health Data Science, Yokohama City University School of Medicine, Yokohama, Kanagawa, Japan; University of Caytania, ITALY

## Abstract

**Objectives:**

This study examined the impact of the Center of Healthy Aging Program (CHAP) on the cognitive function and economic burden associated with dementia.

**Methods:**

This observational study utilised Iwaki cohort data. We included participants with mini-mental state examination (MMSE) scores and categorised them into pre- and post-CHAP groups based on their year of entry into the cohort (before 2013 or after) (index year). We defined participants with suspected severe cognitive impairment and suspected mild cognitive impairment using their MMSE scores, with their incidence being the first observation meeting these definitions during the follow-up period. We compared the incidence rates between the pre- and post-CHAP groups using Cox proportional hazard analysis. Medical and caregiving costs were estimated based on the projected number of residents in Iwaki area with suspected mild cognitive impairment and sSCI during hypothetical 10 years of the CHAP implemented or not and compared.

**Results:**

Of the 2,569 participants, 1716 and 853 were included in the pre- and post-CHAP groups, respectively. The incidence rate of suspected mild cognitive impairment was significantly lower in the post-CHAP group even after adjusted known factors associated with cognitive disorders. No cases of suspected severe cognitive impairment occurred in the post-CHAP group during the follow-up period. Estimated costs of JPY 1,628,450 (USD 11562.00 or EUR 10259.24, JPY 100  =  USD 0.71 or EUR 0.63) and JPY 789,560 (USD 5605.88 or EUR 4974.23) per person per year were projected after 10 years with and without the CHAP, respectively.

**Conclusions:**

We demonstrated a reduction in the incidence rate of suspected mild cognitive impairment among residents who participated in the CHAP and a decrease in the medical and caregiving costs associated with suspected severe cognitive impairment.

## Introduction

Dementia poses a substantial socioeconomic burden on public finances, particularly in middle- and high-income countries facing rapid population aging. In 2015, approximately 47 million individuals had dementia globally, a number projected to escalate to 75 million by 2030 and 135 million by 2050 [1]. Strategies for early prevention include managing one’s lifestyle, reducing stress, addressing vascular risk factors such as hypertension and diabetes mellitus, and managing major depressive disorders [2, 3]. Vascular cognitive impairment (VCI) is the second most common cause of dementia, accounting for 15–20% of dementia cases in North America and Europe and approximately 30% in Asia and some developing countries [4, 5]. Single pathology was reported in approximately 10% of VCI, whereas mixed neurodegenerative and cerebrovascular pathology is more common [4–6]. Besides age, factors preventing VCI and dementia include cardiovascular risk factors such as hypertension, cholesterol, and blood glucose [5, 6]. Recent studies on preventing cognitive decline have emphasised the effectiveness of multi-domain interventions that simultaneously target multiple lifestyle risk factors [7–9]. A recent systematic literature review of randomised controlled trials reported that evidence of the effectiveness of multi-domain interventions is lacking [10].

In Japan, recognised as the world’s most rapidly aging country, 28.8% of the population comprised older adults in 2021 [11]. Consequently, mitigating dementia risk is paramount. To address age-related diseases, Japan offers an annual check-up for all individuals aged 40 years and older, concurrently informing them about disease risks associated with aging and lifestyle habits. While this annual check-up is less expensive than intervention measures and easily implementable, even in rural areas with limited health resources, its impact on behavioural change remains limited, as it is provided only once a year. Therefore, the long-term efficacy of Japan’s nationwide standard check-up remains a subject of debate [12].

The Center of Healthy Aging Program (CHAP), a health programme developed since 2013 in Hirosaki, a rural area in northern Japan, is built on the annual check-up initiated in Iwaki District since 2005 [13] but particularly emphasises raising health awareness to facilitate behavioural change in the entire community in contrast to Japan’s standard check-up (Figure A in S1 Appendix). In particular, the CHAP focuses on accessibility to multiple interventions on wellness and provides programmes across the area to (i) educate health supporters on ways to spread healthy behaviours and social connections, and (ii) increase the opportunity for residents to receive check-ups and their results with personalised explanations and recommendations. This intervention programme is relatively less strong and less expensive than other multi-domain intervention projects [7–10], which allows local governments to implement it; however, the actual effect on health conditions remains controversial. It has been demonstrated that CHAP participants experience a significant long-term reduction in risk factors for cardiovascular diseases, which leads to a future reduction in their incidence and the related medical costs [14]. Several studies have reported an association between these risk factors and the development of dementia [2, 15, 16]. Therefore, the CHAP is considered a potential multi-domain intervention with low costs, but its impact on preventing dementia has not yet been examined.

This study aimed to investigate the effect of the CHAP on cognitive function and estimate the economic burden associated with cognitive impairment under conditions with and without the CHAP.

## Materials and methods

This observational study used data from the Iwaki cohort, which has been collecting annual check-up results since 2005 (Figure B in S1 Appendix) [13, 14]. All participants provided written informed consent. This cohort study adhered to the 1964 Helsinki Declaration and its subsequent amendments, or ethical standards of similar equivalence. It received approval from the Ethics Committee of the Hirosaki University Graduate School of Medicine (2008–025, 2009–015, 2010–020, 2011–033, 2012–050, 2013–062, 2014–014, 2014–377, 2016–028, and 2017–026). This study was registered in the University Hospital Medical Information Network (https://www.umin.ac.jp) prior to the analyses (UMIN ID: UMIN000040459). The protocol for the Iwaki cohort study has been presented in a previous report [13].

### Study population

**Fig 1:**
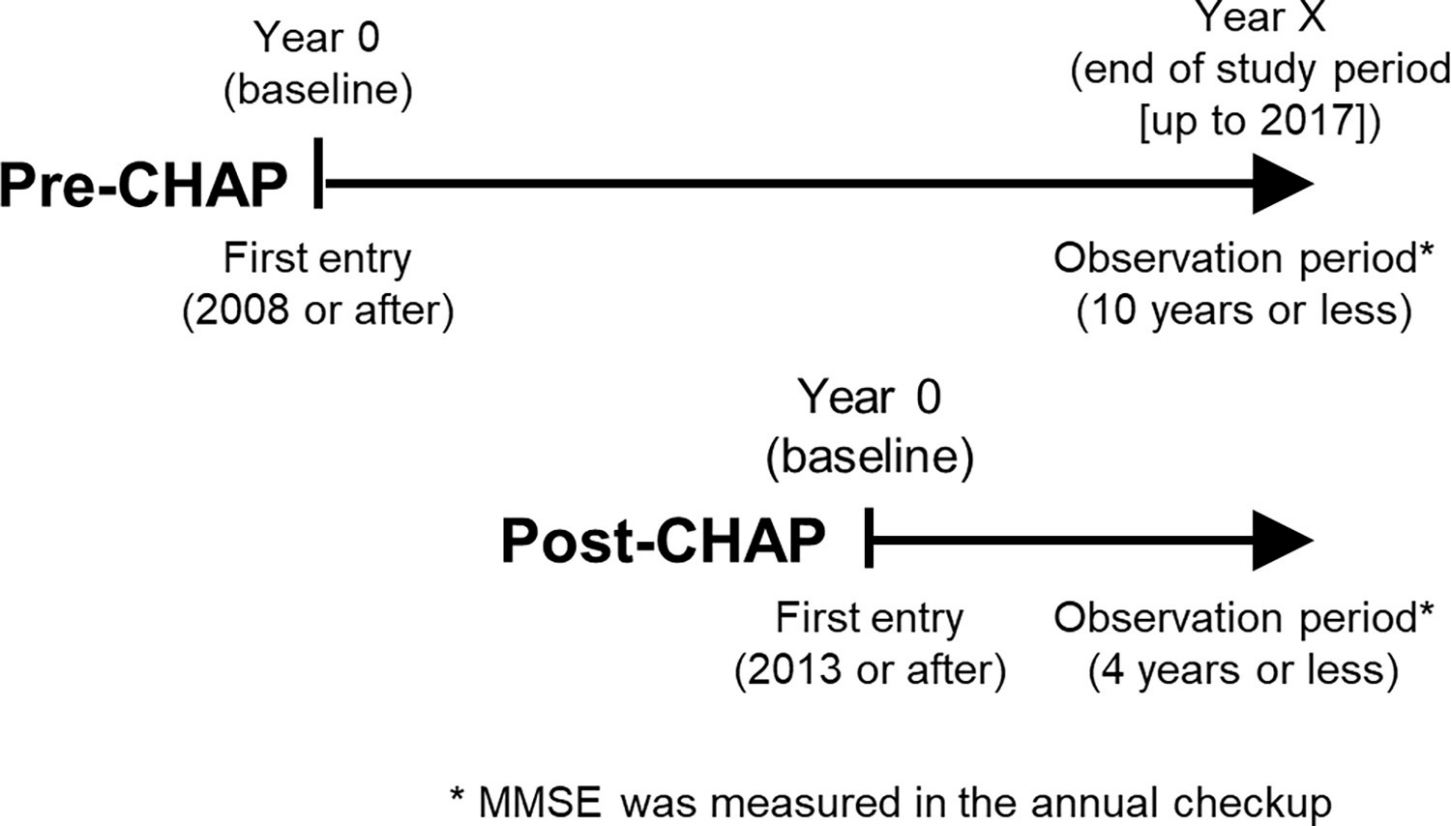
Study scheme.

We used the anonymised data collected after 2008, when the participant proportion exceeded 10% of residents aged 20 years or older in Iwaki, and until 2017, when the mini-mental state examination (MMSE) was last conducted before the COVID-19 pandemic. We accessed the data for the present study purpose on 30 June, 2023. Participants undertook the MMSE during their annual check-up. We included participants with at least one MMSE score measurement and categorised them into pre- and post-CHAP groups based on the year of entry into the Iwaki cohort (index year), that is, before 2013 and 2013 or after, respectively. The former group also had the potential to benefit from the CHAP in 2013 and beyond if they continued to participate in the cohort even after the start of the CHAP. We followed up the included participants from the index year until the earlier date of the year of follow-up (i.e. their last participation) or 2017 (i.e. the end of the study period) (Fig 1).

### Endpoints

The primary endpoint was the incidence rates of suspected severe cognitive impairment (sSCI) and suspected mild cognitive impairment (sMCI) in participants with and without sMCI in the index year, respectively. For this primary endpoint, we defined participants with sSCI and sMCI as having an MMSE score ≤ 23 and 24–27, respectively [17], according to a threshold broadly used by nursing and caregiving providers in Japan, and their occurrence as the first observation of an MMSE score meeting these definitions. We did not correct the MMSE scores according to the participants’ comprehension because of the historically high literacy rates in Japan. We compared the incidence rates between the pre- and post-CHAP groups.

The secondary endpoint involved the reduction in the medical and caregiving costs of a hypothetical cohort within 10 years of the CHAP’s commencement. This was evaluated by comparing the medical and caregiving costs with another hypothetical cohort without access to the CHAP. The hypothetical cohort was assumed to comprise the pre- and post-CHAP groups with identical conditions for every risk factor as those in the index year. Annual costs 10 years later were estimated by multiplying the projected number of participants with sMCI and sSCI by monthly healthcare, social care, and informal care costs per individual with sMCI and sSCI, respectively [18], multiplied by 12. The projected numbers of participants with sMCI and sSCI were derived based on the assumption that the trend in MMSE scores during the observation period from the index year (up to 10 and 4 years for the pre- and post-CHAP groups, respectively), separately calculated for the pre- and post-CHAP groups, as described below, continued for 10 and 4 years in the hypothetical cohort. Finally, annual costs after 10 years were divided by the hypothetical cohort size into annual costs per individual.

For the secondary endpoint, we categorised participants with SCI into mild, moderate, and severe groups using MMSE scores of 21–23, 15–20, and ≤14, respectively, following a previous study reporting the medical and caregiving costs according to the severity of cognitive impairment [18]. As that study classified the mild, moderate, and severe cognitive impairment groups based on mean (SD) MMSE scores of 22.9 (1.6), 17.8 (1.7), and 9.5 (4.5), respectively, we did not estimate the costs of sMCI (defined as an MMSE score of 24–27, which is higher than that reported in the previous study). The medical and caregiving costs reported by this previous study are shown in Table A in S1 Appendix.

### Statistical analysis

Trends in participants’ characteristics from 2008 to 2017 were examined using the Jonckheere–Terpstra trend test. The characteristics of the pre- and post-CHAP groups in the index year were compared using the Wilcoxon test or Fisher’s exact test.

For the primary analysis, to control for differences in characteristics between the pre- and post-CHAP groups, we calculated adjusted hazard ratios using Cox proportional hazard analysis after incorporating known risk factors for cognitive impairment [2, 3, 19–22], including age, sex, the Japanese stroke risk score [23], exercise habits, alcohol intake, hypertension, diabetes, depressive symptoms, and MMSE score in the index year. The Japanese stroke risk score considered other known risk factors, including age, sex, blood pressure, administration of anti-hypertensive drugs, current smoking habits, body mass index, and history of diabetes (indicated by diabetes treatments or fasting blood glucose ≥ 126 mg/dL) [23]. Depressive symptoms were quantified using the Center for Epidemiologic Studies Depression Scale (CES-D). Known risk factors for dementia such as cardiovascular risk factors, aging, and dementia score [24] were not considered because they have not been validated in the Japanese population. Additionally, we measured the adjusted and inverse probability (IP)-weighted hazard ratios. The IP was derived from the propensity score model considering the same risk factors. We excluded the participants who declined to answer questions about alcohol intake and CES-D and had missing values but included them after imputing values in the sensitivity analyses. Multiple imputations were applied to the pooled dataset, generating 100 imputed datasets via predictive mean matching using the duration to onset of sMCI or sSCI, incidence of sMCI or sSCI, age, sex, stroke risk score, exercise habits, alcohol intake, depressive symptoms, and MMSE score. Analyses were performed across all imputed datasets using Rubin’s rules to obtain a set of final estimates.

For the secondary analysis, we used a Poisson generalised linear model. We used the reverse MMSE scores (31 –MMSE score) as the explained variable and considered the aforementioned risk factors to derive the trend in reverse MMSE scores from the index year in both the pre- and the post-CHAP groups. The difference in the observable period between the participants was considered as an offset. From this, we estimated the number of patients with sMCI and sSCI in a hypothetical cohort after a 10-year period with and without the CHAP. Reverse MMSE scores of 31 or more were treated as 30. To ascertain the impact of differences in background characteristics between the groups, we conducted another Poisson generalised linear analysis to directly compare the two groups and adjusted the aforementioned risk factors using the IP weight derived from the same risk factors. The statistical analyses were performed using R software, version 4.2.2 (R Project for Statistical Computing). The statistical tests were two-tailed and a 5% significance level was considered.

## Results

### Patients’ characteristics

Of the 2,569 participants from 2008 to 2017, 1,716 participated before 2013 (pre-CHAP group) and 853 participated in 2013 or later (post-CHAP group). Table 1 presents the baseline characteristics of the pre- and post-CHAP groups. At the index, the post-CHAP group had a significantly lower mean age and Japanese stroke risk score than the pre-CHAP group, but a significantly higher mean MMSE score (Table 1). These findings suggest that residents with lower risk entered the cohort after the CHAP commenced. The MMSE scores (mean ± SD) did not differ between the pre- and post-CHAP groups among participants with sSCI and sMCI (21.611 ± 1.714 vs. 21.000 ± 2.049, p = 0.225 and 25.715 ± 1.049 vs. 25.742 ± 1.085, p = 0.851, respectively), but were slightly higher in the post-CHAP group for those without sMCI (29.468 ± 0.690 vs. 29.613 ± 0.638, p < 0.001). Data on Activities of daily living and instrumental activities of daily living were not collected from this cohort.

**Table 1:** Characteristics of the participants.

	pre-CHAP		post-CHAP		Statistics^a^	p value
	n, mean	%, sd	n, mean	%, sd		
N	1716		853			
Sex (women)	1062	61.90%	496	58.10%	0.856	0.001
Age	54.83	14.92	44.46	15.56	1004866	<0.001
Japanese stroke risk score	20.98	12.96	15.93	13.13	899124	<0.001
BMI	23.06	3.27	22.73	3.93	798949	<0.001
Current smoker^b^	356	20.75%	219^b^	25.70%	1.321	0.005
Alcohol intake^c^	731^c^	42.59%	414	48.53%	1.271	0.005
Depressive symptoms^d^	10.15^e^	6.68	10.69^e^	8.39	555147	0.664
Exercise habits^f^	448	26.10%	209	24.50%	0.919	0.388
MMSE (all)	28.27	2.34	29.22	1.55	545628	<0.001
MMSE (without sSCI^g^)	27.39	1.97	27.24	1.7	12675	0.598
MMSE (without sSCI^h^ or sMCI ^c^)	29.49	0.67	29.57	0.65	188942	0.019

a The Wilcoxon test for continuous variables and Fisher’s exact test were used

b One missing value was excluded

c Two missing values were excluded

d Measured using the Center for Epidemiologic Studies Depression Scale

e 393 and 23 missing values in the pre- and post-CHAP groups, respectively

f Exercise habits mean engaging in exercise that induces sweating two or more times a week for more than 30 min (yes or no), g MMSE ≤ 23

h MMSE 24–27.

MMSE, mini-mental state examination.

The changes in risk factors over time are detailed in Table B in S1 Appendix. The Japanese stroke risk scores increased (i.e., the stroke risk deteriorated) only in the pre-CHAP group, which agreed with our previous report [14]. Depressive symptoms were the worst in year 0 in both the groups (Table B in S1 Appendix). The proportion of current smokers decreased only in the pre-CHAP group and other daily habits did not change during the observation period in either group (Table C in S1 Appendix).

The observable period (mean ± SD) was shorter for those participants with a baseline MMSE score below 20 (1.364 ± 2,540), while it was similar among the participants with other MMSE scores (3.158 ± 3.361, 3.839 ± 3.496, and 3.375 ± 3.356 for MMSE scores of 20–24, 25–29, and 30, respectively).

### Primary analysis

The sMCI incidence rates were 0.044 and 0.024 in the pre- and post-CHAP groups, respectively (Figure C in S1 Appendix). The adjusted odds ratio (OR) was 0.572 (95% confidence interval [CI], 0.365 to 0.897), signifying a significantly lower sMCI incidence rate in the post-CHAP group. The IP-weighted adjusted OR was 0.569 (95% CI, 0.450 to 0.719), supporting this result (Table 2). No cases of sSCI were observed in the post-CHAP group (Table 2) (Figure D in S1 Appendix).

**Table 2:** Incidence rates of sMCI and sSCI.

		N	PY	N of events	Crude IR	Crude OR		Adjusted OR^a^		IP-weighted adjusted OR^b^	
						OR	95% CI	OR	95% CI	OR	95% CI
sMCI	Pre-CHAP	980	5396	235	0.044						
	Post-CHAP	414	915	22	0.024	0.373	0.239–0.583	0.590	0.373–0.934	0.572	0.364–0.898
sSCI	Pre-CHAP	495	2268	67	0.030						
	Post-CHAP	49	93	0.000	0.000	0.000	Inf–Inf	0.000	Inf–Inf	0.000	Inf–Inf

a The covariates used to adjust for the differences in characteristics between the groups were age, sex, Japanese stroke risk score, exercise habits, alcohol intake, Center for Epidemiologic Studies Depression Scale score, and MMSE score at the index. Exercise habits refer to engaging in exercise that induces sweating two or more times a week for more than 30 minutes (yes or no). Alcohol intake refers to drinking alcohol more than several times a month (yes or no). Among the risk factors reported in previous studies [2, 3, 18–21], dietary habits, social isolation, hearing loss, sleep duration, and depressive symptoms were not included owing to insufficient quantification in the Iwaki cohort to distinguish the extent of dementia risk. After imputing missing values, similar ORs (95% CI) were observed (0.572 [0.364–0.898])

b After imputing missing values.

IP, inverse probability; IR, incidence rate; sMCI, suspected mild cognitive impairment; sSCI, suspected severe cognitive impairment; OR, odds ratio; PY, person-year.

### Secondary analysis

The reverse MMSE score decreased significantly by 0.966 and 0.954 per year after adjusting for the risk factors in the pre- and post-CHAP groups and after imputing missing values, respectively. In the pre-CHAP group, age and the reverse MMSE score at baseline were significantly associated with the reverse MMSE score, while in the post-CHAP group, only the reverse MMSE score at baseline showed an association with it (Table 3), which might reflect the difference in baseline characteristics between the groups. The reverse MMSE score reduced significantly, by 0.966 and 0.802, in the pre- and post-CHAP groups when the pre-and post-CHAP groups were directly compared (Table 4).

**Table 3:** Factors related to the MMSE score: Pre-CHAP and post-CHAP^a^.

	Pre-CHAP				Post-CHAP			
	RR	95% CI	Z value	p value	RR	95% CI	Z value	p value
(Intercept)	0.770	0.625–0.948	−2.462	0.014	0.771	0.584–1.018	−1.839	0.066
Sex	0.955	0.884–1.032	−1.163	0.245	1.029	0.905–1.171	0.441	0.659
Age	1.015	1.011–1.019	8.025	<0.001	1.004	0.998–1.010	1.398	0.163
Japanese stroke risk score	1.002	0.998–1.006	1.160	0.246	1.005	0.999–1.012	1.581	0.114
Exercise habits^b^	0.956	0.890–1.027	−1.237	0.216	0.972	0.859–1.101	−0.444	0.657
Alcohol intake^c^	0.939	0.874–1.010	−1.690	0.091	1.014	0.905–1.136	0.232	0.816
Depressive symptoms	1.001	0.996–1.006	0.455	0.649	1.002	0.996–1.009	0.714	0.475
Elapsed years	0.966	0.958–0.975	−7.563	<0.001	0.954	0.916–0.993	−2.309	0.021
Reverse score of MMSE	1.156	1.143–1.168	26.108	<0.001	1.219	1.191–1.247	16.894	<0.001

a Among the risk factors reported in previous studies [2, 3, 18–21], dietary habits, social isolation, hearing loss, sleep duration, and depressive symptoms were not included owing to insufficient quantification in the Iwaki cohort to distinguish the extent of dementia risk

b Exercise habits mean engaging in exercise that induces sweating two or more times a week for more than 30 min (yes or no)

c Alcohol intake refers to drinking alcohol more than several times a month (yes or no).

MMSE, mini-mental state examination; RR, risk ratio

**Table 4:** Factors related to the MMSE score: Pre-CHAP vs. post-CHAP using the IP-weighted model^a^.

	RR	95% CI	Z value	p value
(Intercept)	0.820	0.691–0.973	−2.272	0.023
Post-CHAP (ref = pre-CHAP)	0.835	0.779–0.895	−5.123	<0.001
Sex	0.971	0.909–1.037	−0.890	0.374
Age	1.012	1.009–1.016	7.934	<0.001
Japanese stroke risk score	1.003	0.999–1.006	1.679	0.093
Exercise habits^b^	0.966	0.908–1.028	−1.096	0.273
Alcohol intake^c^	0.945	0.890–1.004	−1.840	0.066
Depressive symptoms	1.002	0.998–1.006	0.900	0.368
Elapsed years	0.966	0.957–0.974	−7.940	<0.001
Reverse score of MMSE	1.166	1.155–1.178	30.877	<0.001

a Among the risk factors reported in previous studies [2, 3, 18–21], dietary habits, social isolation, hearing loss, sleep duration, and depressive symptoms were not included owing to insufficient quantification in the Iwaki cohort to distinguish the extent of dementia risk

b Exercise habits mean engaging in exercise that induces sweating two or more times a week for more than 30 min (yes or no)

c Alcohol intake refers to drinking alcohol more than several times a month (yes or no).

MMSE, mini-mental state examination; RR, risk ratio

**Fig 2:**
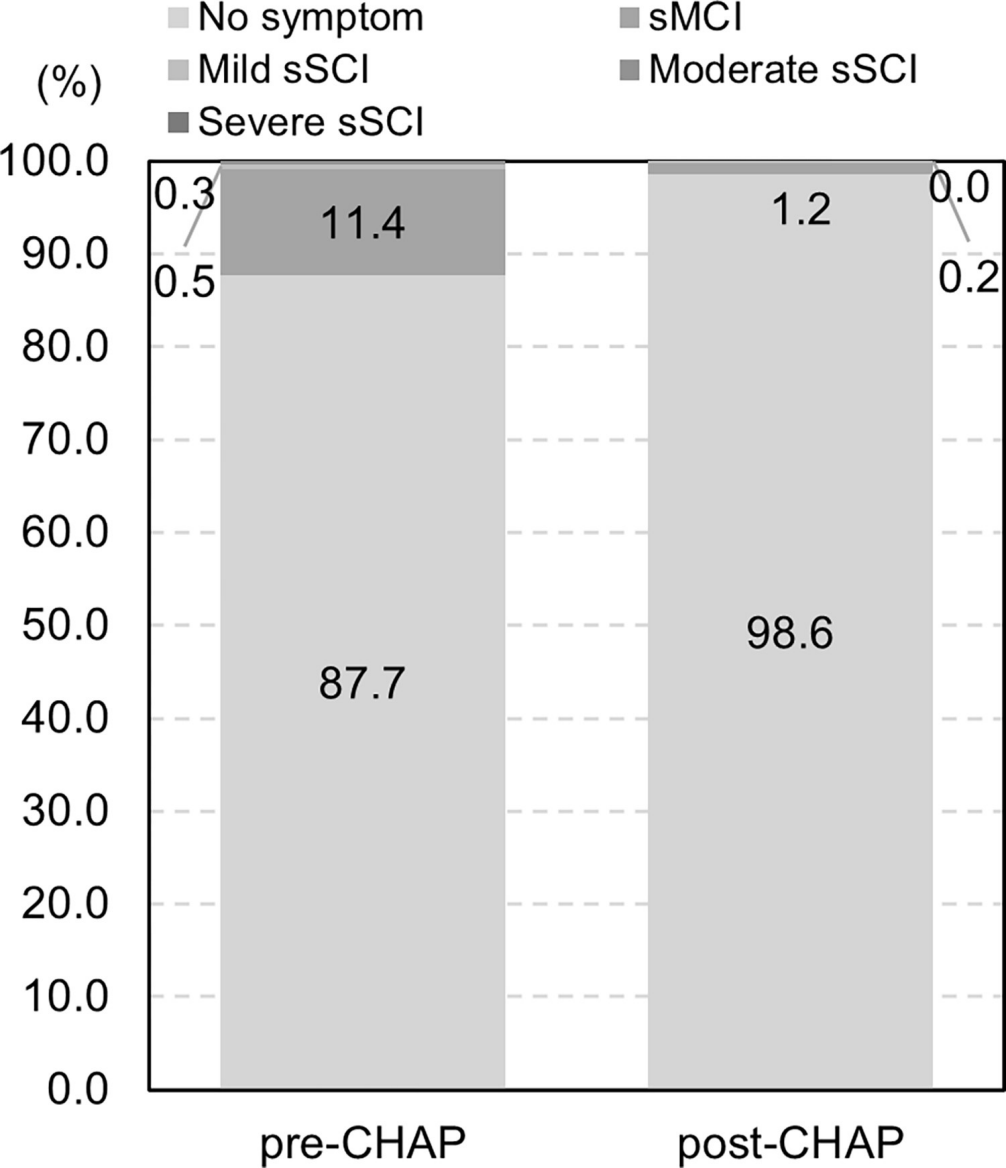
Predicted proportion of individuals with sMCI and sSCI^a^. a No severe sSCI was projected in either group. sMCI, suspected mild cognitive impairment; sSCI, suspected severe cognitive impairment.

**Fig 3:**
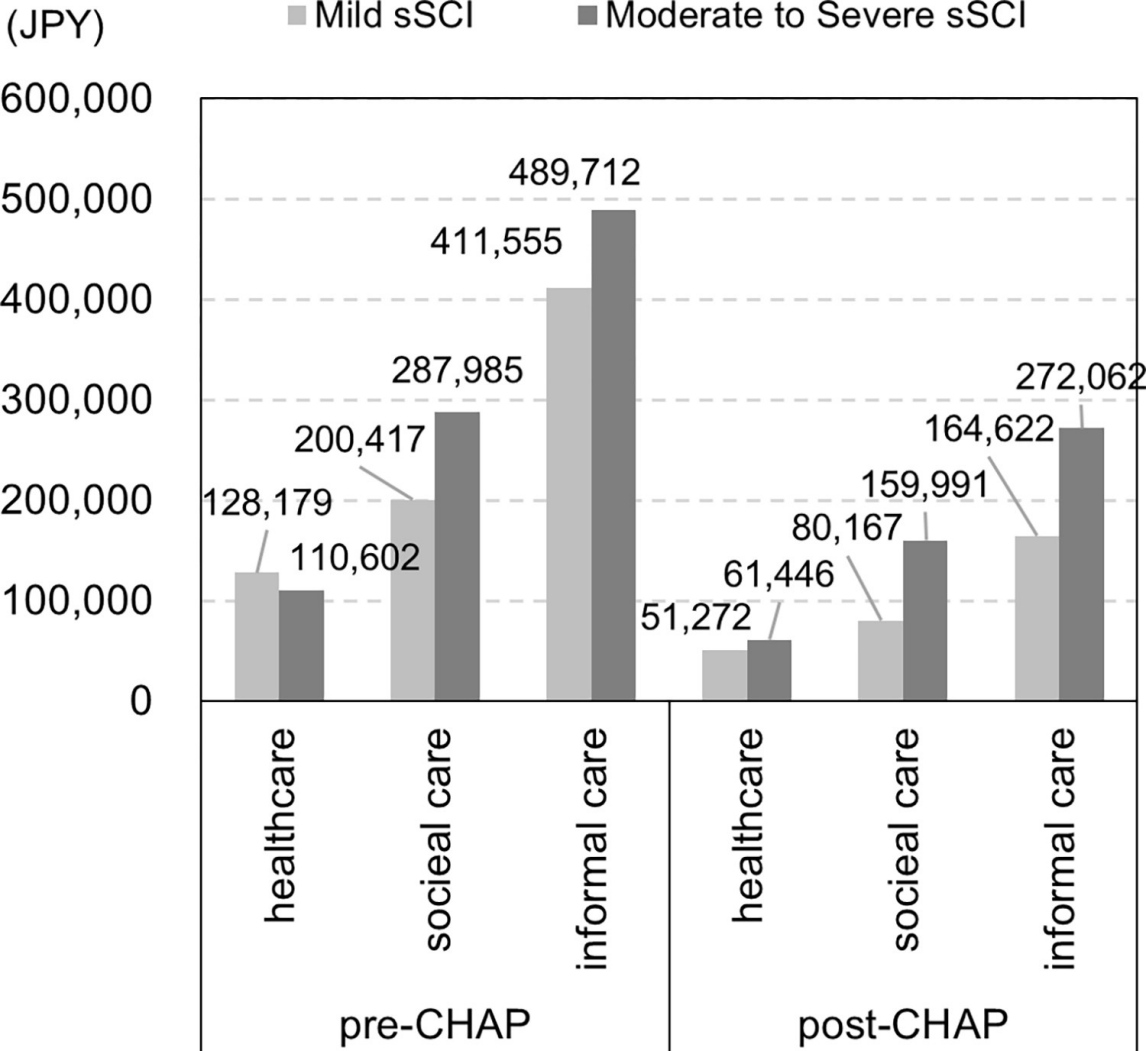
Estimated costs associated with sSCI. sMCI, suspected mild cognitive impairment; sSCI, suspected severe cognitive impairment.

We used the model directly comparing the pre- and post-CHAP groups under the assumption that the trend of the reverse MMSE score continued after the index. We found that 10.1% and 6.5% of the participants were predicted to suffer sMCI in a hypothetical cohort of 2,659 residents after 10 years without and with the CHAP, respectively; by contrast, 0.7% and 0.4% of the participants were predicted to suffer sSCI (Fig 2). The total costs associated with SCI were estimated to be JPY 1,628,450 (USD 11562.00 or EUR 10259.24, JPY 100  =  USD 0.71 or EUR 0.63) and JPY 789,560 (USD 5605.88 or EUR 4974.23) per person per year in the hypothetical cohort after 10 years without and with the CHAP, respectively, corresponding to more than a 50% reduction per year (Fig 3). Using the separate model for the pre- and post-CHAP groups, the total costs after 10 years with and without the CHAP were JPY 5,282,495 (USD 37505.71 or EUR 33279.72) and JPY 370,076 (USD 2627.54 or EUR 2331.48) per person per year, respectively, equivalent to a more than 90% reduction per year.

## Discussion

We showed that the CHAP associated with lower incidence rates of sMCI and sSCI based on four years of observations following its commencement, even though the programme does not include a strong intervention for directly altering participants’ behaviour and cognitive function. This reduction is linked to the potential future decreases in the number of residents who need healthcare resources and thus in the medical and caregiving costs associated with sSCI. This supports that the CHAP encourages positive changes in cognitive function-related factors. In particular, we observed a deterioration in the Japanese stroke risk score in the pre-CHAP group but no change in the post-CHAP group. On the other hands, we found no improvement in daily habits in the post-CHAP group, which might be associated with the low number of participants enrolled and the short observation period after the start of the CHAP, overly rough classification of alcohol intake and exercise habits (yes or no), and lack of information on dietary habits. Previous reports have suggested that certain dietary components and patterns may delay cognitive decline [25–30]. To clarify the impact of the CHAP on healthy behaviours, additional analyses must be carried out after gathering longer-term data enough to observe the effect of behavioural changes on cognitive function.

The main characteristic of the CHAP, a relatively less strong multi-domain intervention aiming to facilitate simultaneous changes in multiple health-related behaviours, is its low cost. Previous multi-domain intervention studies have shown that strong interventions in dietary habits, exercise, and cognitive training independently and significantly mitigate risks of cognitive impairment [31]. On the contrary, the CHAP aims to improve health awareness by providing participants with specific information about their health conditions and associated risks immediately after their annual check-up, followed by broad information thereafter [32]. Additionally, in the CHAP, volunteers support residents’ engagement in healthy behaviours and locations for confirming their health conditions are offered throughout the Hirosaki region. Retail stores are recommended to expand the selection of healthy diets. A previous study showed that cardiovascular disease risk indicators significantly improved after the CHAP’s implementation, with no change in the proportion of individuals using therapeutic drugs [14]. In the present study, we found another impact on cognitive function.

The most important finding of our study is that the CHAP holds value for every community, especially as budget constraints tighten owing to the increasing risk of dementia associated with the aging population. One previous review indicated that in rural and remote areas such as those in Australia, resources for public health interventions are limited and accessing healthcare services can be challenging. However, the economic burden of dementia is growing significantly owing to the more rapid aging in these areas than in urban regions [33]. Similar regional disparities have been observed in Japan and other Western countries [34–36]. Interventions with lower costs and a certain effectiveness, such as the CHAP based on annual check-ups, are deemed valuable in such areas. Additionally, a community-based approach is crucial for reducing the incidence of dementia at the population level regardless of whether in rural or urban areas, because it does not require the identification of high-risk individuals and can promote automatic changes in habits [37].

### Limitations of this study

First, our findings are based on only one region in Japan; therefore, their generalisability to other Japanese regions and globally must be confirmed in future studies. Second, our results may not necessarily reflect the effects of the programmes themselves owing to concerns about unmeasured confounders (e.g. risk dietary habits that we could not include in the present study), given the limited information available to control for such confounding factors. Third, we relied on medical and caregiving costs per patient, categorised by the level of cognitive impairment, based on a previous study. This was because the Iwaki cohort data did not include information on healthcare resource utilisation. However, dementia often coexists with other age-related diseases, leading to variations in dementia care and healthcare resource utilisation among individuals. We categorised participants into two and four cognitive impairment groups according to their MMSE scores (i.e. sMCI and sSCI/sMCI as well as mild, moderate, and severe sSCI) in the primary and secondary analyses, respectively, but the severity of cognitive impairment does not necessarily correlate with this score; this may have caused the over- or underestimation of costs after 10 years. Future analyses using comprehensive healthcare records from individuals, encompassing medical treatment, nursing care, and other data such as local government claims databases, are essential for a more sophisticated evaluation of the CHAP’s effectiveness. Fourth, we hypothesised that the difference in MMSE scores between the groups could be attributed to improved health awareness, but we were unable to confirm this owing to the lack of a measurement index such as health literacy in the Iwaki cohort. We hope to establish an increase in health awareness following the CHAP’s implementation in the future. Fifth, although a previous report has indicated an association between MMSE scores and age and educational level [38], we considered age in the multivariate analyses but did not correct the MMSE scores according to the participants’ educational background. Because no resident showed an MMSE score < 10, we believe that the impact of a low MMSE is limited, even without correction.

## Conclusions

This study demonstrated a reduction in the incidence rate of sMCI among residents who participated in an innovative health awareness programme. Additionally, the findings indicated a decrease in medical and caregiving costs associated with sSCI in a rural area of Japan.

## Supporting information

S1 Appendix (DOCX)
