# Supplementary material for: Reduction in the incidence of cognitive impairment and related costs through an innovative health awareness programme in rural Japan
Source: PLoS One. 2024 Oct 14;19(10):e0311826. doi: 10.1371/journal.pone.0311826 (PMC11472916; doi:10.1371/journal.pone.0311826)
Supplement: S1 Appendix — (DOCX) [file pone.0311826.s001.docx]

**S1 Appendix**

**Table A. Costs by the class of MMSE score reported in the previous study**

| Cognitive  impairment | MMSE score | | Average monthly costs, JPY (USD, EUR) | | | |
| --- | --- | --- | --- | --- | --- | --- |
|  | Mean | SD | healthcare | Social care | Informal care | Total |
| Mild sSCI | 22.9 | 1.6 | 27441  (194.83, 172.88) | 42906  (304.63, 270.31) | 88107  (625.56, 555.07) | 158454  (1125.02, 998.26) |
| Moderate sSCI | 17.8 | 1.7 | 26309  (186.79, 165.75) | 68503  (486.37, 431.57) | 116488  (827.06, 733.87) | 211300  (1500.23, 1331.19) |
| Severe sSCI | 9.5 | 4.5 | 26649  (189.21, 167.89) | 91731  (651.29, 577.91) | 175845  (1248.50, 1107.82) | 294225  (2089.00, 1853.62) |

sSCI, suspected severe cognitive impairment

**Table B. Trends in clinical characteristics after the index^a^**

| Year of follow-up | pre-CHAP | | | | | post-CHAP | | | | |
| --- | --- | --- | --- | --- | --- | --- | --- | --- | --- | --- |
|  | n | Risk score of stroke | | Depressive symptom | | n | Risk score of stroke | | Depressive symptom | |
|  |  | Mean | SD | n | % |  | Mean | SD | n | % |
| year 0 | 1716 | 20.98 | 12.96 | 10.15 | 6.68 | 853 | 15.93 | 13.13 | 10.69 | 8.39 |
| year 1 | 923 | 21.09 | 12.36 | 8.83 | 6.61 | 345 | 15.82 | 13.02 | 9.48 | 7.86 |
| year 2 | 898 | 21.47 | 12.32 | 7.45 | 7.03 | 259 | 16.11 | 12.50 | 9.97 | 8.25 |
| year 3 | 817 | 22.53 | 12.94 | 9.25 | 7.91 | 172 | 14.84 | 12.12 | 9.76 | 8.56 |
| year 4 | 811 | 23.09 | 13.15 | 8.91 | 8.18 | 77 | 15.30 | 13.08 | 9.64 | 8.81 |
| year 5 | 740 | 23.76 | 13.17 | 8.86 | 7.52 | 0 | - | - | - | - |
| year 6 | 706 | 25.15 | 12.22 | 8.86 | 7.40 | 0 | - | - | - | - |
| year 7 | 610 | 24.38 | 12.04 | 9.63 | 7.41 | 0 | - | - | - | - |
| year 8 | 511 | 25.81 | 11.65 | 9.58 | 7.64 | 0 | - | - | - | - |
| year 9 | 381 | 25.23 | 11.15 | 9.22 | 7.01 | 0 | - | - | - | - |
| Statistics^a^ |  | 15868825 | | 8048532 | |  | 486110 | | 442760 | |
| p value |  | 0.002 | | 0.002 | |  | 0.780 | | 0.012 | |

a Jonckheere-Terpstra test and Cochran-Armitage trend test were used for continuous variables and categorical variables, respectively,

**Table C. Trends in clinical daily habits after the index^a^**

| Year of follow-up | pre-CHAP | | | | | | | post-CHAP | | | | | | |
| --- | --- | --- | --- | --- | --- | --- | --- | --- | --- | --- | --- | --- | --- | --- |
|  | n | Current smoking | | Alcohol intake^b^ | | Exercise habits^c^ | | n | Current smoking | | Alcohol intake^b^ | | Exercise habits^c^ | |
|  |  | n | % | n | % | n | % |  | n | % | n | % | n | % |
| year 0 | 1716 | 356 | 20.75 | 731 | 42.59 | 448 | 26.11 | 853 | 219 | 25.70 | 414 | 48.53 | 209 | 24.50 |
| year 1 | 923 | 146 | 15.82 | 404 | 43.74 | 290 | 31.38 | 345 | 93 | 26.96 | 173 | 50.14 | 97 | 28.12 |
| year 2 | 898 | 125 | 13.92 | 359 | 39.98 | 299 | 33.30 | 259 | 68 | 26.25 | 121 | 46.72 | 73 | 28.19 |
| year 3 | 817 | 111 | 13.62 | 347 | 42.47 | 265 | 32.44 | 172 | 42 | 24.42 | 65 | 38.01 | 40 | 23.26 |
| year 4 | 811 | 113 | 13.93 | 330 | 40.74 | 262 | 32.31 | 77 | 20 | 25.97 | 40 | 51.32 | 23 | 29.87 |
| year 5 | 740 | 94 | 12.70 | 320 | 43.22 | 235 | 31.76 | 0 | - | - | - | - | - | - |
| year 6 | 706 | 82 | 11.61 | 308 | 43.63 | 229 | 32.44 | 0 | - | - | - | - | - | - |
| year 7 | 610 | 69 | 11.31 | 254 | 41.71 | 178 | 29.18 | 0 | - | - | - | - | - | - |
| year 8 | 511 | 60 | 11.76 | 222 | 43.44 | 152 | 29.75 | 0 | - | - | - | - | - | - |
| year 9 | 381 | 37 | 9.71 | 160 | 42.11 | 117 | 30.71 | 0 | - | - | - | - | - | - |
| Statistics^a^ |  | 60.185 | | 0.022 | | 3.338 | |  | 0.009 | | 2.197 | | 0.671 | |
| p value |  | <0.001 | | 0.881 | | 0.068 | |  | 0.924 | | 0.138 | | 0.413 | |

a Jonckheere-Terpstra test and Cochran-Armitage trend test were used for continuous variables and categorical variables, respectively, b Alcohol intake refers to drinking alcohol more than several times a month (yes or no), c Exercise habits mean engaging in exercise that induces sweating two or more times a week for more than 30 min (yes or no).

**Figure A. Location of Aomori Prefecture, Hirosaki City (light blue), and Iwaki District (blue)**

This figure was created by authors using digital national land information which Ministry of Land, Infrastructure, Transport and Tourism in Japan provides.


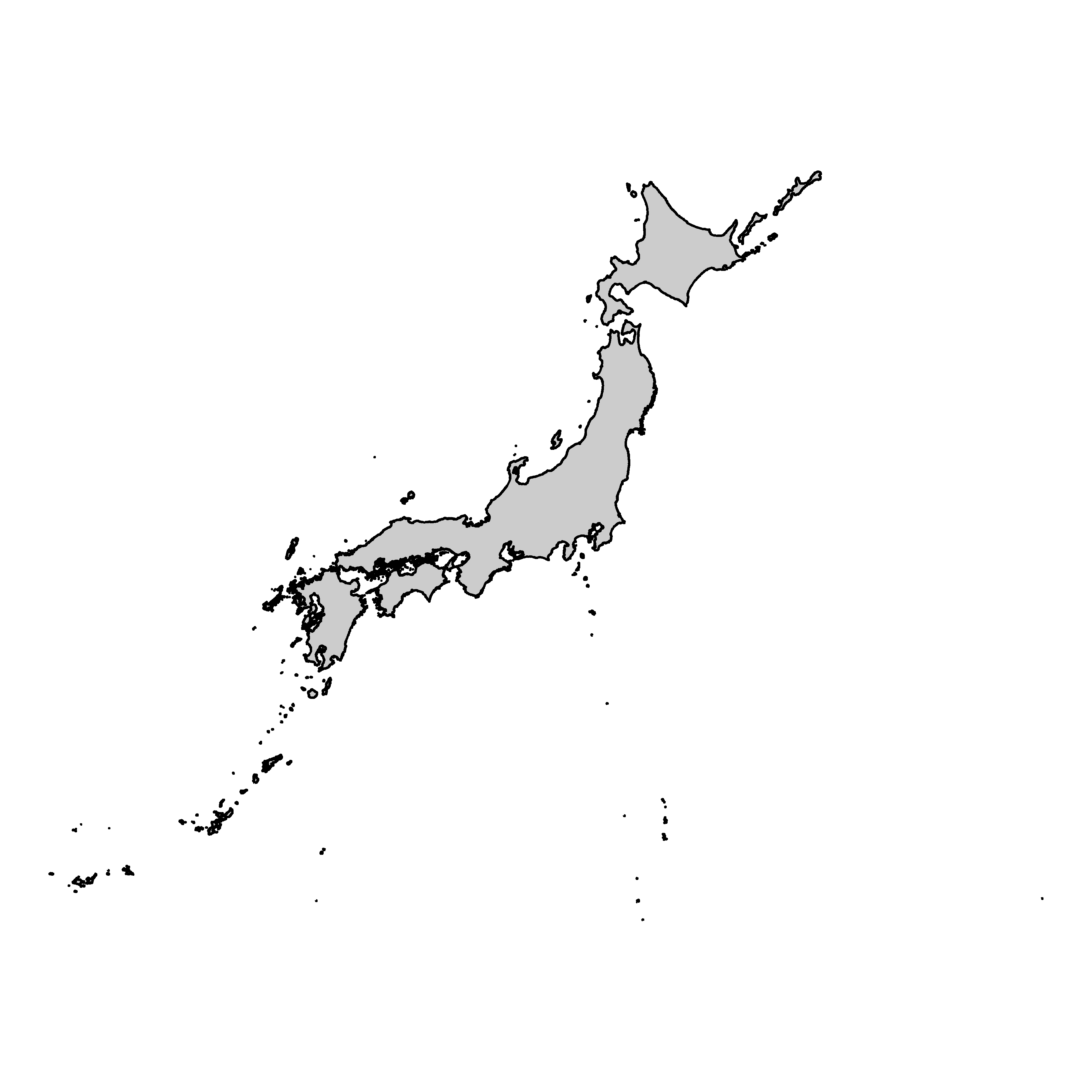

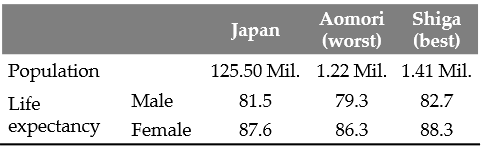

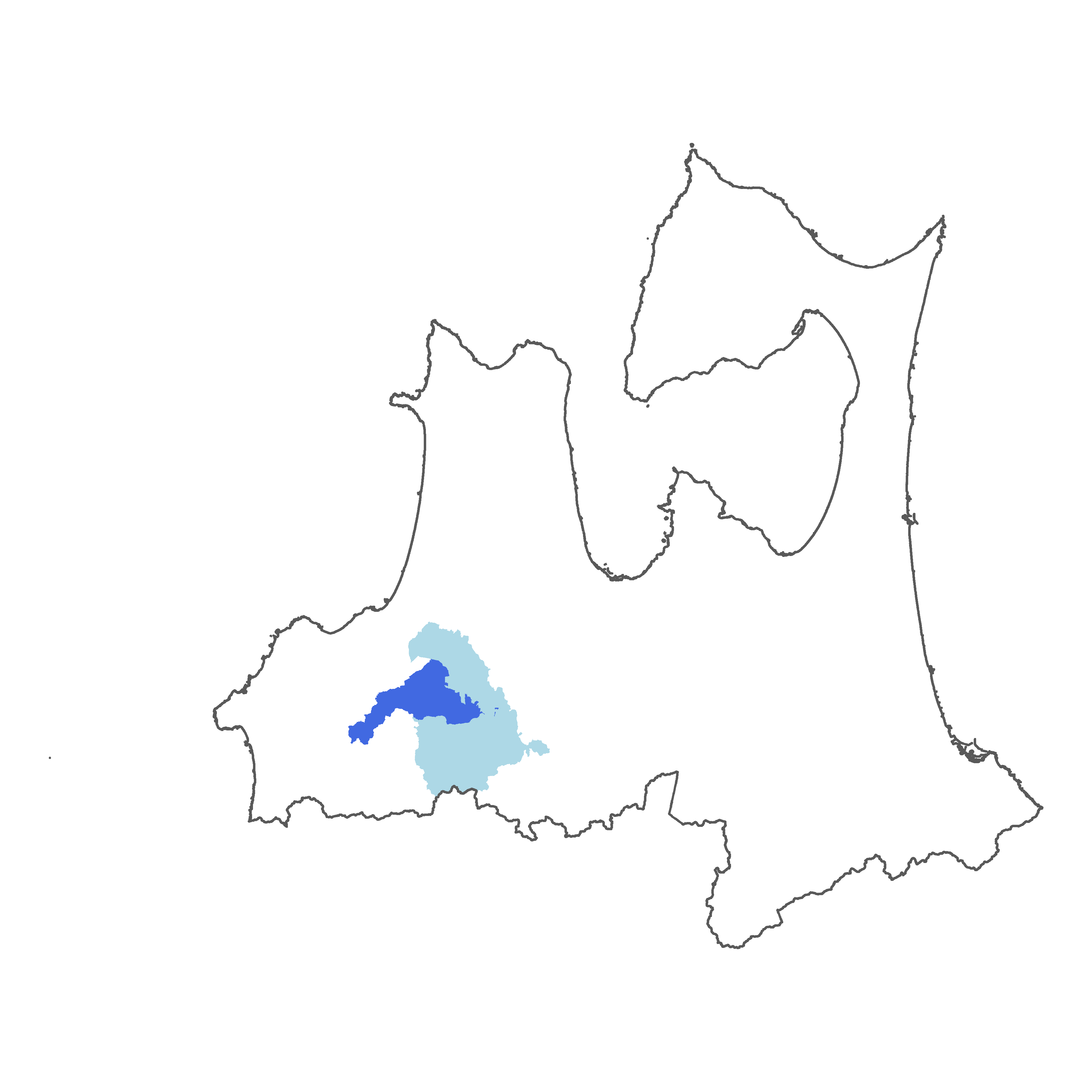


**Figure B. The Period and Coverage of Iwaki Cohort Data. Iwaki district is located in Aomori Prefecture, and Iwaki cohort data contains checkup results before and after the start of CHAP.**

**
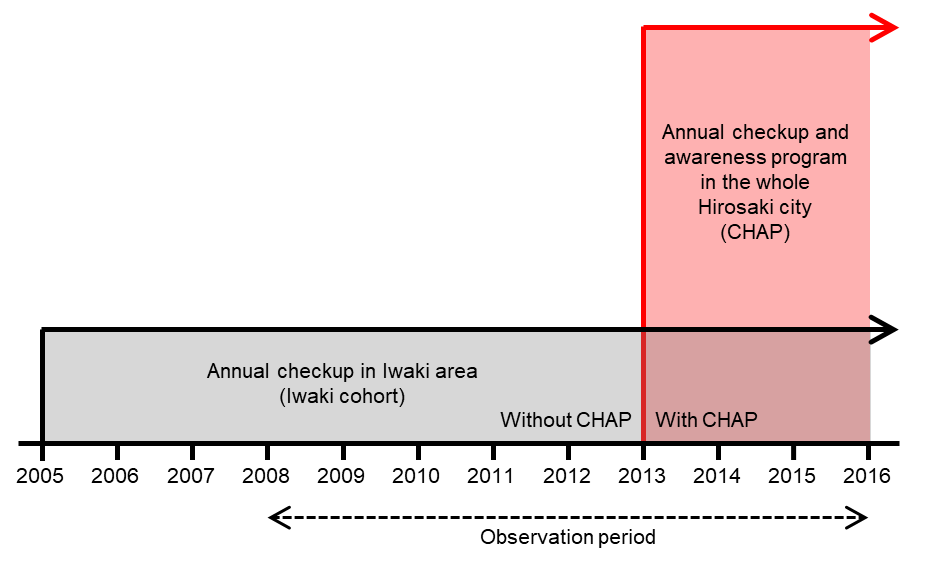
**

CHAP, Center of Healthy Aging Program.

**Figure C. Cumulative incidence of sMCI^a^ in subjects without sMCI and sSCI at the baseline**


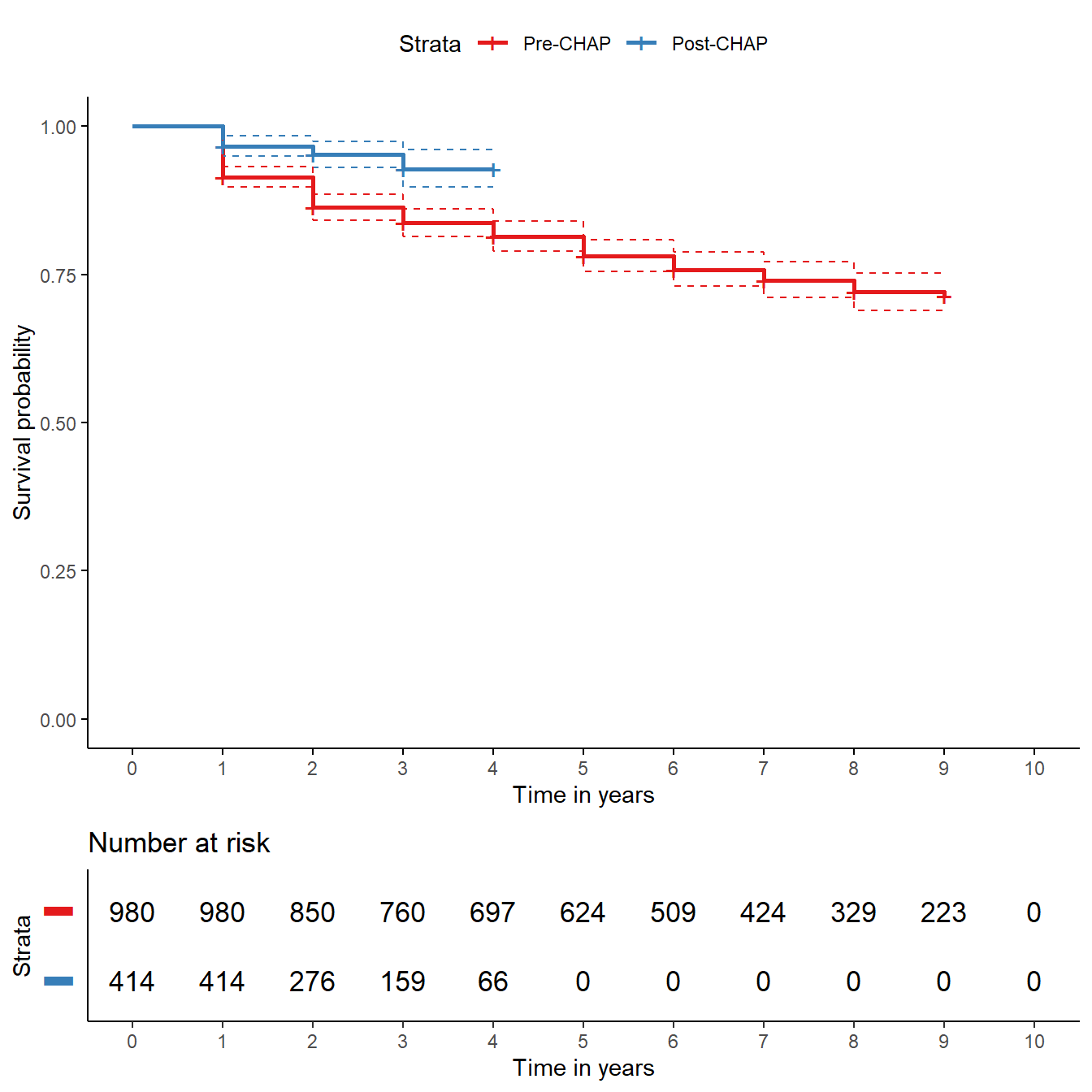


**Figure D. Cumulative incidence of sSCI^b^ in subjects with sMCI and without sSCI at the baseline**


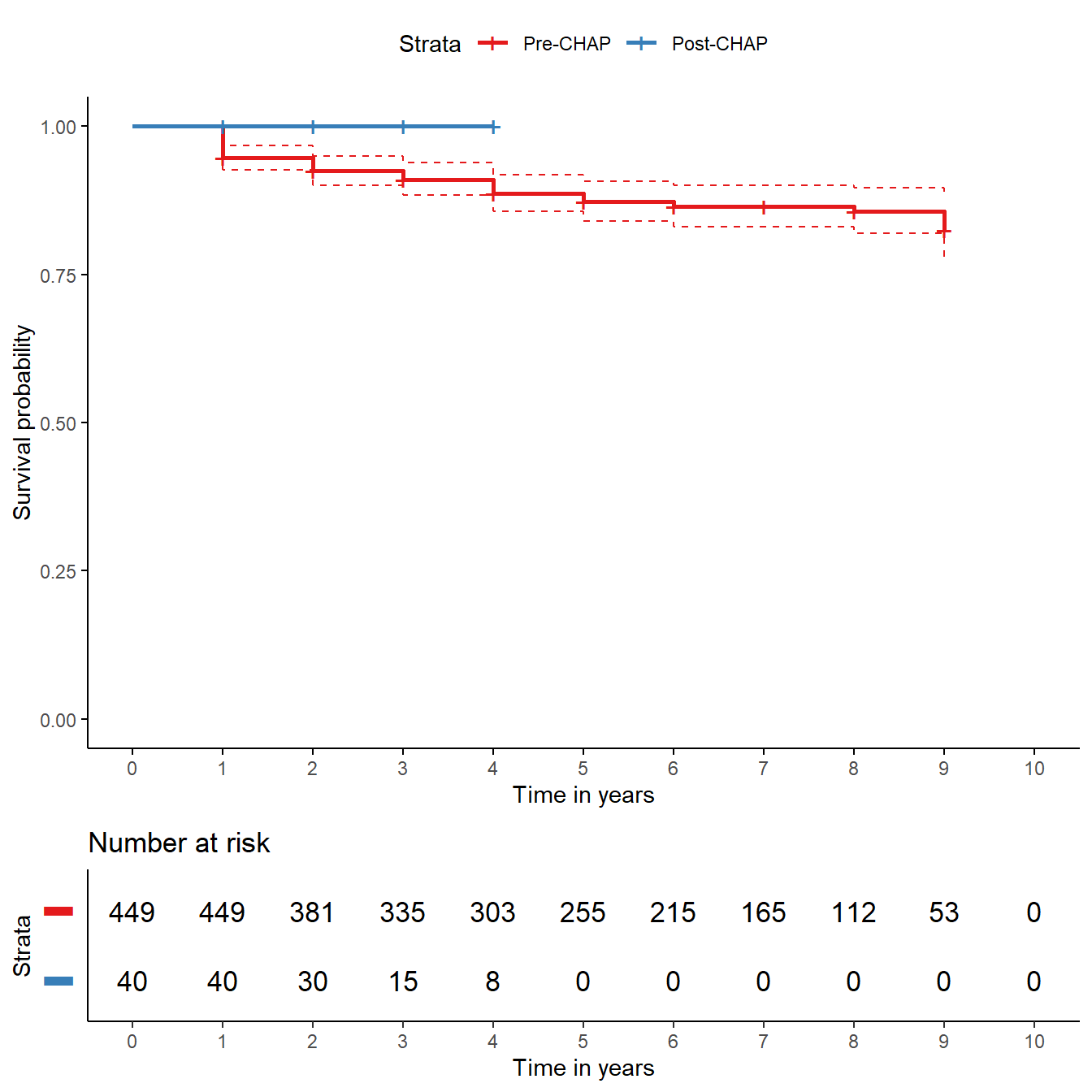


a MMSE 24≤ 27, b MMSE ≤ 23

MMSE, mini-mental state examination; sMCI, suspected mild cognitive impairment; sSCI, suspected severe cognitive impairment
